# Negative Allosteric Modulators of A_2A_R: A New Weapon for Cancer Immunotherapy?

**DOI:** 10.1021/acs.jmedchem.5c00137

**Published:** 2025-01-30

**Authors:** Alfonso Zambon

**Affiliations:** Department of Chemical and Geological Sciences, University of Modena and Reggio Emilia, Modena I-41125, Italy

## Abstract

Adenosine-mediated activation of A_2A_R drives immunosuppressive signaling in high-adenosine tumor microenvironments (TMEs), impeding anticancer immunity. Targeting A_2A_R with negative allosteric modulators (NAMs) is a promising approach for cancer immunotherapy: unlike the orthosteric antagonists currently in use, which face competitive and off-target limitations, NAMs leverage a noncompetitive, saturable mechanism that enhances receptor selectivity. The development of a novel series of A_2A_R NAMs demonstrates potent activity within high-adenosine TMEs, underscoring a significant translational potential in oncology.

Adenosine is an endogenous purine nucleoside that mediates a wide variety of cellular functions by binding with four G-protein-coupled receptors, G_i_-coupled A_1_R and A_3_R and G_s_-coupled A_2A_R and A_2B_R, each of which has a distinct pharmacological profile.^1^ The adenosine-A_2A_R pathway in particular mediates an anti-inflammatory response and plays an important role in protecting normal organs and tissues from the autoimmune response of immune cells by activation of adenosine cyclase and production of intracellular cAMP, but its activation by accumulation of extracellular adenosine promotes cancer immune escape. Indeed, a high adenosine concentration in the tumor microenvironment (TME) correlates with tumor aggressiveness, and blocking A_2A_R can inhibit the progression of a variety of solid tumors. Negative modulation of A_2A_R is thus an attractive approach in the context of tumor immunotherapy.^2^

Orthosteric drugs bind at the active site of the target protein, competing with the natural ligand; in presence of high concentration of this ligand, such as that encountered in high-adenosine TME, an orthosteric drug might face competition and could be less effective at lower doses. Indeed, the repurposing as cancer immunotherapies of orthosteric antagonists of A_2A_R designed to work at low adenosine concentrations has shown modest benefit in early trials, although, encouragingly, significant progress has been achieved recently by combining second-generation orthosteric antagonists of A_2A_R with immune checkpoint inhibitors.^3^ Nevertheless, the widespread expression and ubiquity of the adenosine receptors poses a challenge to the development of safe and potent therapies involving A_2A_R blockade, as these can lead to adverse side effects, especially in normal tissues where adenosine plays critical roles in regulating cardiovascular, inflammatory, and immune responses. Moreover, the doses required to achieve therapeutic efficacy in a high-adenosine TME may be too high, leading to toxicity.^4^

Conversely, allosteric modulation involves the binding of a ligand at a site distinct from the orthosteric site. This results in a conformational change that alters the receptor’s activity in a more subtle and selective manner: allosteric modulators act by changing ligand binding cooperativity, i.e., affecting the binding of orthosteric ligands to the target protein. Allosteric modulators can then either enhance (positive allosteric modulators, or PAMs) or inhibit (negative allosteric modulators, or NAMs) receptor function without directly competing with the natural ligand. Allosteric modulation has emerged as a powerful approach in drug discovery, as it offers significant advantages over orthosteric modulation with respect to both selectivity and modulation of effect. The advantages of allosteric modulation lie in its ability to fine-tune the receptor’s activity and provide more precise control over its signaling, which can be beneficial for therapeutic purposes. Importantly, allosteric modulators are not competitive, and their mode of action is saturable; i.e., they reach maximum efficacy once all allosteric sites are occupied and do not depend on the concentration of the natural ligand. Negative allosteric modulation is thus characterized by a decrease in the affinity and/or the efficacy of orthosteric ligands, including the natural ligand(s) of a receptor. This saturable effect allows NAMs to work at the minimum efficacious dosing of a treatment, as increased dosage does not result in increased therapeutic effect, limiting the risk of toxicity. Off-target side effects of allosteric ligands are also expected to be lowered, as allosteric pockets are postulated to be less conserved with respect to the orthosteric binding site, and hence it should be possible to achieve higher receptor selectivity.^5^ Efficacious NAMs of A_2A_R, scantly reported so far, have then the potential of provide a safer, efficacious therapy option for cancer immunotherapy on high-adenosine TME.^6^

The Featured Article “Discovery of the First-Efficacious Adenosine 2A Receptor Negative Allosteric Modulators for High Adenosine Cancer Immunotherapies” in this issue of the *Journal of Medicinal Chemistry* describes the identification and optimization of a series of A_2A_R antagonists with a confirmed NAM mode of action, leading to the identification of several potent compounds which retain activity in a high-adenosine environment and potentially paving the way for the development of first-in-class drugs for cancer immunotherapies.^7^ Reporting the identification, testing, and optimization of these compounds, the authors highlight some interesting medicinal chemistry themes in the development of NAMs and of allosteric modulators in general.

Two of the main issues in the development of allosteric modulators are the lack of structural information allowing the identification of allosteric sites, which hinders the *in silico* identification of potential hits, and the requirements of functional assays that enable the identification of molecules that affect target function irrespective of the site of binding.^5^ In this Featured Article, the team carried out a high-throughput screening campaign aimed at the identification of allosteric modulators on a relatively small, manually curated library of drug-like compounds using a cellular assay specifically developed in-house; this assay allows the monitoring of intracellular cAMP, which is elevated after triggering of the cAMP/PKA/CREB pathway by A_2A_R activation, in presence of a set concentration of adenosine. Importantly, by measuring a downstream biomarker, this assay does not affect binding to the target receptor, ruling out any possible interference with any putative allosteric sites. This approach allowed for the efficient identification of several A_2A_R antagonists, exemplified by compound **1**, whose NAM mode of action was confirmed by the same assay. The allosteric mode of action of the series was further proved by showing that compounds **4** and **5**, identified by scaffold hopping from **1**, have an effect on ligand binding cooperativity, reducing the association rate and increasing the dissociation rate of an orthosteric ligand. The team then carried out a streamlined medicinal chemical effort from the initial hits, taking advantage of the chemical tractability of the dicyanopyridine chemical scaffold of **4**, which gave easy access to a library of over 50 compounds in 2 or 3 synthetic steps. Remarkably, this was sufficient to elucidate the structure–activity relationship (SAR) of the series and to identify a set of compounds with increased potency and maximum response, selectivity against the control cell line and other adenosine receptors, and a 1000-fold improvement in both binding cooperativity α and dissociation constant *K*_B_ (Figure 1).

**Figure 1:**
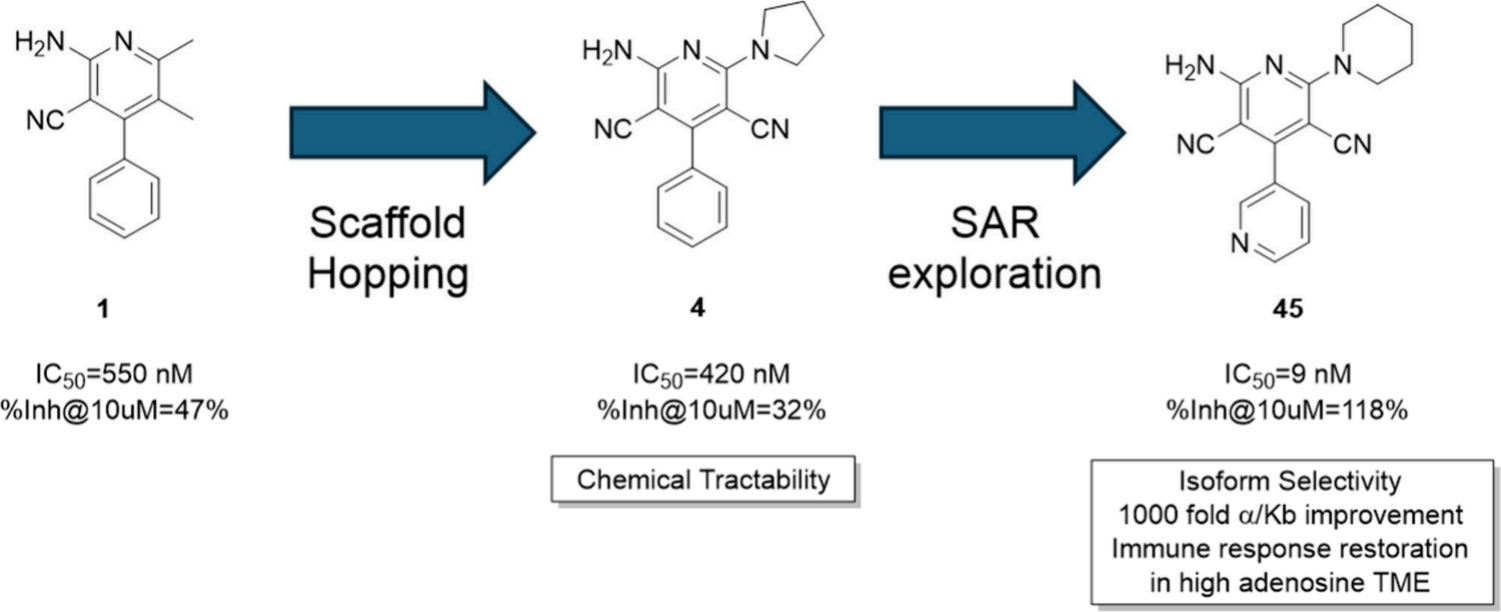
Evolution of efficacious A_2A_R NAM **45** from initial hit **1**.

Profiling of the most advanced compounds showcases the distinct features of NAMs with respect to those of orthosteric ligands. All compounds confirm a saturable mode of action; this is crucial toward their application on tumors characterized by high-adenosine TME, as this effect bears little dependence on the concentration of the natural ligand. Indeed, with adenosine concentration ranging from 20 nM to 3 μM, selected compounds such as **28** and **48** show a <10-fold loss of activity against a roughly 50-fold loss of the orthosteric clinical candidate Imardent (AZD4635). Most promisingly, this advantage is carried over when assessing as a biomarker the downstream phosphorylation of CREB (pCREB) in CD4^+^ T lymphocytes, which express a high level of A_2A_R, under highly immunosuppressive conditions. The orthosteric antagonist Imaradenant caused only partial restoration of immunomodulatory activity, while optimized compounds such as **28**, **44**, and **48** were able to increase pCREB levels in a dose-dependent manner, reaching full recovery at 1 μM. This suggests the potential to induce both partial and full immune response in high-adenosine TMEs in translational settings.

Intriguingly, allosteric modulators seem to occupy a chemical space slightly distinct from that of orthosteric compounds: allosteric modulators have a propensity to be smaller, more lipophilic, and rigid than their corresponding orthosteric ligands, and they tend to have favorable physicochemical properties, which may be orthogonal to those of the natural mediator.^8^ Furthermore, as mentioned above, allosteric sites tend to be less homologous than orthosteric sites among related proteins.^5,9^ This provides the opportunity to gain a strong IP position, even in the highly competitive field of A_2A_R antagonists. In this program, the authors were able to file a composition of matter patent in a crowded area such as that of the dicyanopyridine scaffold (WO2023213761A1),^10^ setting the ground for its progression toward the late lead optimization stage and preclinical development. This is crucial, as the most advanced compounds presented show high permeability but moderate solubility (class II BCS drugs) and short half-life upon incubation in human and mouse liver microsomes. Improvement of the pharmacokinetic properties of the lead candidates is likely to be needed before translation of the series to the clinic.

This Featured Article provides a fine example of how challenging targets can be tackled in innovative ways by exploiting alternative modes of actions that make it possible to overcome issues encountered by orthosteric compounds. Remarkably, the team was able to develop very rapidly compounds with favorable potency and selectivity and to give proof of target engagement in high-adenosine settings, despite the lack of any structural information. This was possible thanks to a streamlined medicinal chemistry campaign on chemically tractable early hits underpinned by an *ad-hoc, in-house* assay that allowed for the rapid elucidation of their mode of action. In summary, the identification of NAMs of A_2A_R represents an exciting step toward the development of novel agents for safer immunotherapies in specific tumor settings, and the series has the potential for a fast progression toward the clinic.
